# The impact of an ‘evergreening’ strategy nearing patent expiration on the uptake of biosimilars and public healthcare costs: a case study on the introduction of a second administration form of trastuzumab in The Netherlands

**DOI:** 10.1007/s10198-023-01648-w

**Published:** 2024-01-08

**Authors:** Ghyli Kirshner, Peter Makai, Chiara Brouns, Lonneke Timmers, Ron Kemp

**Affiliations:** 1 NZa, Utrecht, The Netherlands; 2 Erasmus University Rotterdam, Rotterdam, The Netherlands; 3 ACM, P.O. Box 16326, 2500 BH The Hague, The Netherlands; 4 Zorgverzekeraars Nederland, Zeist, The Netherlands; 5 National Healthcare Institute, Diemen, The Netherlands

**Keywords:** Biosimilar, Competition, Pricing, Evergreening, I18, I11, L12

## Abstract

In this paper, we explore dynamic market share and public healthcare costs of trastuzumab’s evergreening (subcutaneous) variant during introduction of trastuzumab’s competitive biosimilar variants in the Netherlands. We used a time series design to assess dynamic market share of trastuzumab’s evergreening variant after introducing trastuzumab’s biosimilar variants, focusing on the number of treatments and patients. The public healthcare costs of this evergreening strategy were estimated using administrative claims data. Our results show that the original trastuzumab was completely replaced by the subcutaneous and biosimilar variants. The uptake of the subcutaneous form peaked at 50% market share but after the introduction of biosimilars progressively reduced to a market share of 20%, resulting in a more competitive market structure. The public healthcare costs for trastuzumab significantly decreased after the introduction of the biosimilars. After the introduction of the biosimilars, a substantial price drop is visible, with the subcutaneous version, still under patent, also falling sharply in price but less strongly than the iv/biosimilar version. As the costs are publicly funded, we recommend a more explicit societal debate to consider if the potential benefits of subcutaneous Herceptin^®^ (and other similar medicines) are worth the additional costs, and at which price it should be reimbursed as the part of the benefit package.

## Introduction

Globally, major concerns exist on the cost of (expensive) medicines which put pressure on total healthcare expenditures. Since the expenditures on these medicines increase more rapidly than other care, there is the risk of crowding out other healthcare services [1]. The high expenditures are to a large extent due to the monopoly prices set by pharmaceutical companies as their medicines are protected by patents [2]. Patents for original pharmaceuticals are typically valid for 20 years [3], with the possibility to extend the patents with 5 years in case of a lengthy development time and successful market authorization [4]. After patent expiration, other pharmaceutical companies can enter the market with a generic (chemical molecule) or biosimilar (biological medicine). Biosimilars and generics are normally offered at lower prices and allow for price competition as the pharmaceutical company no longer has a monopoly.

By 2018, 34 biological drugs have become available off-patent and in the next few years, 15 more biological drugs will reach the end of their market exclusivity 4 [5]. In European countries, biosimilar list price savings (excluding savings from confidential rebates and discounts) accounted for €5.7 billion in 2020 [6]. As biosimilars bring budgetary relief to healthcare payers, the lower drug costs can also lead to an increase in treatments [6, 7], i.e., more patients can be treated using the same budget resulting in lower total budget savings but also more health gain.

Given the beneficial position of the pharmaceutical company during the patent term, pharmaceutical companies have an incentive to engage in strategies to prolong the period of patent protection of their drugs and their monopoly power. One of the strategies is secondary patenting or so-called ‘evergreening’ in which pharmaceutical companies extend the drug’s exclusivity period. They do so by filing additional patents on the already patent-protected drug, shortly before the initial patent expires, by making (minor) modifications to the existing drug [8]. Some of the best-selling drugs have large patent portfolios and are protected by more than a hundred patents [9]. This creates a high barrier for generics and biosimilars to enter the market after the initial patent of the branded drug has expired [10].

An example of a drug subject to evergreening is trastuzumab for breast cancer. Trastuzumab is an immunotherapeutic medicine that is used in treatments for patients with HER2 + early and metastatic breast cancer. Trastuzumab was first registered as Herceptin^®^ by Roche in 2000 as an intravenously administered drug. In 2013, Roche received authorization for a newly patented subcutaneous administration form of trastuzumab, the evergreening version. This was just several months before the patent on the intravenous administration form expired in 2014. In 2018, the first intravenous administration form of biosimilars received authorization to enter the market, the competitive version. Trastuzumab is not the only drug for which a pharmaceutical company introduced a subcutaneous administration form near patent expiry (Table 1).

**Table 1:** Biological drugs and their dates of patent expiry for the IV product in Europe and approval of SC product, and biosimilars by EMA

Reference product	Patent expiry IV	Approval SC	Approval first biosimilar
Rituximab (Mabthera^®^)	November 2013	March 2014	February 2017
Trastuzumab (Herceptin^®^)	July 2014	July 2013	November 2017
Tocilizumab (RoActemra^®^)	April 2017	April 2014	N.A
Abatacept (Orencia^®^)	December 2017	October 2012	N.A
Natalizumab (Tysabri^®^)	February 2023	April 2021	N.A

This list is not exhaustive

*IV* Intravenous administration form, *SC* subcutaneous administration form

The efficacy and safety are similar for all administration forms. On the other hand, the different administration forms might be preferable form different viewpoints (patient- or hospital preferences). As successful evergreening can lead to foregone societal loss, it is important to assess the impact of the evergreening strategy on the uptake of biosimilars like trastuzumab (see Section “Pharmaceutical market structure & patent loss”). When pharmaceutical companies succeed in prolonging their drug’s exclusivity period by introducing another administration form and succeed in keeping prices high, savings on biosimilars are limited. In this article, we cover the gap in the literature by exploring the dynamic market share and public healthcare costs of trastuzumab’s evergreening (subcutaneous) variant during introduction of trastuzumab’s competitive biosimilar variant in the Netherlands. We perform this exercise from a health insurance perspective (payer’s perspective).

## Theoretical framework

### Pharmaceutical market structure and patent loss

To stimulate the investment and innovation of new drugs, the pharmaceutical market operates under a patent system. Since R&D costs can be extremely high, few companies would be willing to risk significant investment without the assurance of getting a patent [11]. From the day the patent application is submitted, patent protection has a duration of twenty years. After the patent application, it takes several years to complete the research and development of the drug and obtain FDA/EMA approval, leaving on average 12.4 years of market exclusivity [12]. Specifically for the Dutch market, a recent study found market exclusivity for 11.3 years [13]. The pricing of the newly entered drug is influenced by the presence (or lack) of therapeutic alternatives on the market and the perceived added societal value of the drug, which is an important factor in the society’s willingness to pay for the drug [14, 15].

As soon as the patent protection of a biological drug expires, biosimilars are allowed to enter the market. As they compete with the reference drug, they often must make themselves attractive by entering the market at a significantly reduced price compared to the reference drug. Lower prices are partially possible due to fewer necessary investments in R&D and lower manufacturing costs [3]. More importantly though, prices of the reference drugs often bear little relationship to R&D costs but are more often value based, which leads to extremely high prices for the reference drug [16]. The entrance of biosimilars will create a competitive market structure in which prices for both the reference drug and biosimilars are significantly lower compared to the price(s) before biosimilar entry [17].

Given the lower price benefits of biosimilars entry for the sustainability of healthcare systems, health authorities in different countries have implemented policies to promote the uptake of biosimilars. As a result, there are European countries where certain biosimilars have obtained almost 100% of the market shares [18, 19].

As an attempt to retain their market shares, originator, patent-holding pharmaceutical companies often have a strategy near patent expiration to prolong the lifecycle of the drug [10]. In the US, originator companies can prolong the protected status by introducing their own generic, as the first-filing generic in the US. is granted 180 days market exclusivity. Pay-for-delay settlements are patent settlements in which the company pays the potential generic competitors to delay market entry. In 2016, 11% of the patent settlements in Europe showed value transfers from the originator company to the generic company to limit generic market entry [20]. These types of settlements are often under scrutiny with the antitrust laws [21]. With secondary patenting, a pharmaceutical company files for an additional patent on features other than the original active drug ingredient. Such patents could be filed on different formulations, alternative forms of molecules, compositions, dosing, packaging, or administration route of the originator [2, 22]. There are two important differences between generics and biosimilars, in the requirements for market approval. Generics are chemically equivalent to the originator and can immediately access the market. In contrast, biosimilars require an additional clinical trial to show the safety, quality and effectiveness of the biosimilar to be comparable to the original product [23]. Although generics and biosimilars are allowed to enter the market once the original patent has expired, the adjusted branded drugs are often already widely used by the patient population which makes it more difficult for biosimilars to effectively penetrate the market. Concern has risen over the years regarding whether these evergreening strategies restrict market competition, keep drug costs unnecessarily high and thus threaten access to medicines [2, 24, 25]. For example, the pharmaceutical company Abbott Laboratories succeeded in staving off competition for its drug fenofibrate by sequential launching of branded reformulations. It is estimated that this strategy costs the US healthcare system $700 million annually [26].

### The case of trastuzumab

In this study, we will use trastuzumab as a case study, one of the first biological drugs where the patent expired and an evergreening strategy was used. HER2 + breast cancer, for which trastuzumab is used, is observed in 20%–30% of all breast cancers [27, 28]. Early-stage breast cancer patients receive trastuzumab in addition to chemotherapy and subsequently as monotherapy for one year after the first administration. Metastatic breast cancer patients also receive trastuzumab directly as monotherapy if previous chemotherapy has failed [29], in addition to the regimen of early-stage breast cancer patients. Trastuzumab significantly improves survival outcomes for women with HER2-overexpressing breast cancer [30].

Trastuzumab was brought on to the European market under the name Herceptin® by the pharmaceutical company Roche in August 2000 and was included in the Dutch basic healthcare package in 2005. It entered the market as an intravenously administered drug [29]. The patent for this intravenous administration form expired in Europe in July 2014. For trastuzumab’s subcutaneous administration form, Roche received authorization by the EMA in July 2013. Since it is therapeutically equivalent, the subcutaneous form was automatically included in the Dutch basic healthcare package and the administration form was first used in the Dutch hospitals in 2014. The intravenous administration takes up 30–90 min and subcutaneous administration 5 min. Considering the time difference, the subcutaneous administration time is perceived to be more patient friendly and relieves pressure on the capacity of the hospital’s oncology day care units [31]. There are also differences when administered in combination with chemotherapy or as monotherapy. When patients receive chemotherapy, they need an intravenous line and trastuzumab can then easily be administered intravenously as well. The subcutaneous administration form is preferred as monotherapy as no intravenous line is required. Moreover, a subcutaneous administration form is more suitable for treatment at home than intravenous. However, a Dutch study showed that home-based subcutaneous treatment is more costly than hospital-based subcutaneous treatment [32].

In the Netherlands, hospitals provide both inpatient care requiring an overnight stay as well as outpatient specialist care not requiring an overnight stay for oncology patients, for example administering an infusion. Dutch hospitals usually negotiate with pharmaceutical companies and purchase the medicines themselves. They also negotiate with health insurers^1^ about the price they claim for (1) the treatment including the administration of drugs, and (2) the price of the expensive medicines, which are billed as an additional reimbursement as a so-called add-on at the insurer. This bargaining is an important feature of the systems, meaning that if a hospital can achieve higher reimbursement than its costs, potential savings do not have an impact on the public healthcare costs [33]. Therefore, in this study, we define public healthcare costs as the costs made by the health insurers for the trastuzumab medicine. In other words, we use a payers’ perspective, which reflects the price, that is paid by Dutch citizens. The price paid by hospitals to pharmaceutical companies to purchase the drug is confidential and might be different. It should be noted that the declaration code for administering drugs is used for intravenously administered drugs as well as for subcutaneously administered drug. From health insurers (payers’) perspective, the price will be the same in both situations. We, therefore, focus on the costs of the drug trastuzumab itself. In 2018, trastuzumab had the seventh highest expenditure of all medicines in the Netherlands [1].

As the patent for the intravenous trastuzumab expired in 2014, biosimilars were allowed to enter the market. Since the patent for the subcutaneous administration form is valid until 2030, only intravenous trastuzumab biosimilars can enter the market [34]. In June 2018, the first biosimilar, Herzuma^®^, entered the Dutch market, after which Kanjinti^®^, Ogivri^®^, Trazimera^®^, Ontruzant^®^, and Zercepec^®^ followed, decreasing Roche’s market share and resulting in a competitive market structure [35].

However, the uptake of trastuzumab biosimilars might not be as high as it would have been without the monopoly on the subcutaneous administration form. Hospitals invested in the switch from intravenous Herceptin^®^ to subcutaneous Herceptin^®^, switching back to intravenous biosimilars means that they would again have to invest money and time to implement the use of another administration form. Patients need to be informed and instructed and the accompanied administrative tasks can be substantial [36]. Moreover, it was pointed out that the acceptance of patients is higher when they switch from an intravenous reference drug to an intravenous biosimilar than from a subcutaneous administration form to an intravenous biosimilar [36]. Therefore, hospitals might encounter resistance of patients who are treated with subcutaneous trastuzumab because they prefer this administration form and might perceive the intravenous biosimilar as a different (and maybe less effective) drug.

## Research methods

### Data and variable construction

For this study, we used proprietary insurance claim data of all patients who were treated with trastuzumab in Dutch hospitals between January 2013 and December 2020. The dataset of trastuzumab claims consists of 347,106 claims for 18,809 patients, each claim representing one treatment for breast cancer.^2^ All patients, those with and without simultaneous chemotherapy, are included in the analysis. Claims in which patients receive multiple administrations with different administration forms on the same day (*n* = 224) are excluded because this is assumed to be an administrative mistake. Furthermore, claims with multiple package sizes of the same brand of treatment on the same day are merged (*n* = 8987), resulting in 337,915 distinct claims for 18,809 patients. We aggregated the data per month on a hospital level resulting in 6044 observations.

Based on the trastuzumab brand used for the treatment, the observations are classified as intravenous Herceptin^®^, subcutaneous Herceptin^®^, or biosimilar (any brand). We converted the number of packages for intravenous Herceptin^®^ and biosimilars into milligrams. The dosage of intravenous trastuzumab is 6 mg/kg, so dosages vary among patients. Subcutaneous trastuzumab is used in a fixed dosage of 600 mg, irrespective of patients’ weight. The age of the patient is defined as the age in years on the day the patient received the treatment. ‘Simultaneous chemotherapy’ is a binary variable (0 = no simultaneous chemotherapy, 1 = simultaneous chemotherapy). We defined simultaneous chemotherapy as follows: trastuzumab’s administration date falls within ± 3 days of the administration date of intravenous chemotherapy. The add-on claims for the chemotherapy drugs docetaxel and paclitaxel are used, since these are indicated to be given in combination with trastuzumab. Hospitals are divided into one of the following categories: university-based, top clinical or general hospital. Lastly, we included insurance companies in the analysis. Insurance companies apply different policies to reduce drug costs, including encouraging the use of biosimilars. The largest insurer will likely have the most impact with their preference policy on the medicine policy of the hospital. We used dummy variables to identify the largest insurer within the hospital. We defined separate dummy variables for the four largest health insurers and used the combined six smaller health insurers as the reference group. Besides the dummy, we also included the market share of this largest insurer as an indication for the strength of its negotiation position and impact on the hospital medicine purchasing strategy.

### Empirical strategy

We studied the effect of introducing biosimilars on the use of subcutaneous trastuzumab using a single-center interrupted time series (ITS) design 3 as we are interested in the development of subcutaneous trastuzumab use over time and not merely at a specific cut-off point [37]. Data from January 2014 (introduction subcutaneous form) up to and including December 2020 are used in the regressions. All hospitals are assigned to the treatment at the same time, 01–06–2018, because the probability of receiving the treatment changes exactly from 0 to 1 after this introduction date of the biosimilar [35, 36]. The following regression equation is used^4^,^5^,^6^:

$${Y}_{{\text{it}}}= \alpha + {\beta }_{0}{X}_{{\text{it}}}+ {\beta }_{1}{r}_{{\text{it}}}+ {\beta }_{2}{r}_{{\text{it}}}^{2}+ {\beta }_{3}{r}_{{\text{it}}}*{X}_{{\text{it}}}+ {\beta }_{4 }{r}_{{\text{it}}}^{2}* {X}_{{\text{it}}}+ {C}_{{\text{it}}}+ {\nu }_{i}+{\varepsilon }_{{\text{it}}} .$$

Outcome $Y$ is the proportion of subcutaneous trastuzumab use by a hospital $i$ in month $t$. The introduction of the biosimilars is a binary variable ${X}_{{\text{it}}}$ with value 0 if $t$ < 01–06–2018 and value 1 if $t$ ≥ 01–06–2018. ${r}_{{\text{it}}}$ is the rating variable which is centered on the cut-off point (${r}_{{\text{it}}}$–cut-off score), which locates the intercept at the cut-off point. Interactions between ${X}_{{\text{it}}}$ and ${r}_{{\text{it}}}$ account for a possible change in the intercept as well as different effects in slope on both sides of the cut-off points [38]. The covariates ${C}_{{\text{it}}}$ include the mean age of the patients, the size of the hospital (the number of patients treated with trastuzumab), the dominant health insurer, and the market share of the dominant health insurer.

Additionally, to assess whether the introduction of biosimilars had a significant impact on the total number of trastuzumab treatments, the number of patients per month and the mean dosage (in milligrams), we performed single-center ITS analyses with aggregated data per month on a national level. The following regression is used^7^:

$${Y}_{t}= {\beta }_{0}+ + {\beta }_{2}{X}_{t}+{\beta }_{1}{T}_{t}+ {T}_{t}^{2}+ {\beta }_{3}{T}_{t}* {X}_{t}+{\beta }_{4}{T}_{t}^{2}*{X}_{t}+ {C}_{t}+ {\varepsilon }_{t} .$$

Outcome $Y$ is total number of trastuzumab treatments, the number of patients or the mean dosage per month $t$. The introduction of the biosimilars is a binary variable ${X}_{t}$ with value 0 if $t$ < 01–06–2018 and value 1 if $t$ ≥ 01–06–2018, which shows the immediate effect of the introduction of biosimilars on the total number of treatments, the number of patients or the mean dosage. ${T}_{t}$ is the time since the start of the study which is January 2014. ${X}_{t*}{T}_{t}$ estimates the difference in trend before and after biosimilar introduction. ${C}_{t}$ is a dummy variable with value 0 if $t$ < 01–03–2020 and value 1 if $t$ ≥ 01–03–2020 to control for the effect of the Covid-19 pandemic on the supply of healthcare.^8^

Lastly, we estimated the additional reimbursement costs of subcutaneous Herceptin^®^. From June 2018 up until December 2020, the monthly difference in the mean costs between subcutaneous Herceptin^®^ and biosimilars was multiplied with the proportion of subcutaneous Herceptin^®^ and the total number of treatments in that month.

## Results 

### Descriptives of use

The descriptive statistics are presented in Table 2. The mean number of treatments per month is 3519.95. The mean number of treatments per hospital per year is 580.61 and for all years 4628.97. The mean number of milligrams administered per intravenous Herceptin^®^ treatment is 410.48 and for biosimilars 451.99. For subcutaneous Herceptin^®^, a fixed dosage of 600 mg is used. 99.67% of the patients are female and the mean age of patients is 56.78 years.

**Table 2:** Descriptives of trastuzumab use concerning 18,809 patients and 73 hospitals over 8 years (2013–2020)

	*M*	SD	Min	Max
Number of treatments per month	3519.95	250.04	2814	4009
Number of treatments per hospital 2013–2020	4628.97	2940.64	1095	19,653
Number of treatments per hospital per year	580.61	379.87	34	2771
Milligrams per treatment				
Herceptin IV^®^	410.48	172.06	0.3	3000
Herceptin SC^®^	600.00	0	600	600
Biosimilars	451.99	135.71	0.42	1710
Age of patients	56.51	11.87	17	97
% female patients	99.67	5.74	0	1

*M* Mean, *SD* standard deviation, *IV* intravenous administration form, *SC* subcutaneous administration form

The treatments were given in a total of 73 hospitals in the Netherlands, of which 8 are academic hospitals (3,143 treatments and 1,507 patients per year on average), 25 are top clinical hospitals (20,372 treatments and 9,226 patients per year on average), and 40 are general hospitals (18,725 treatments and 8,274 patients), see Table 3.

**Table 3:** Descriptives of trastuzumab use per hospital type

	Academic	Top clinical	General
Number of hospitals	8	25	40
Number of patients 2013–2020	1507	9226	8274
Number of treatments per year	3,142.63 (348.45)	20,372.13 (1032.35)	18,724.63 (936.16)

Standard deviation in parentheses

On average in the treatment cycle of a patient, 4.80 treatments with trastuzumab are given in combination with chemotherapy whereas 13.16 treatments are given without chemotherapy (Table 4). Of all intravenous-administered trastuzumab treatments (Herceptin^®^ and biosimilars), 33.13% is given in combination with chemotherapy. For subcutaneous administered trastuzumab, this is 9.74%, mainly caused by subcutaneous-only hospitals. These hospitals do not offer intravenous trastuzumab as a treatment option and therefore these patients receive trastuzumab subcutaneously next to the intravenously administered chemotherapy.

**Table 4:** Descriptives of chemotherapy use in combination with trastuzumab treatments

	*M*	SD	Min	Max
Number of trastuzumab treatments with chemotherapy	4.80	4.39	0	50
Number of trastuzumab treatments without chemotherapy	13.16	13.10	0	251
% of intravenous trastuzumab treatments with chemo	33.13	47.07	0	1
% of subcutaneous trastuzumab treatments with chemotherapy	9.74	29.65	0	1

*M* Mean, *SD* standard deviation

**Fig. 1:**
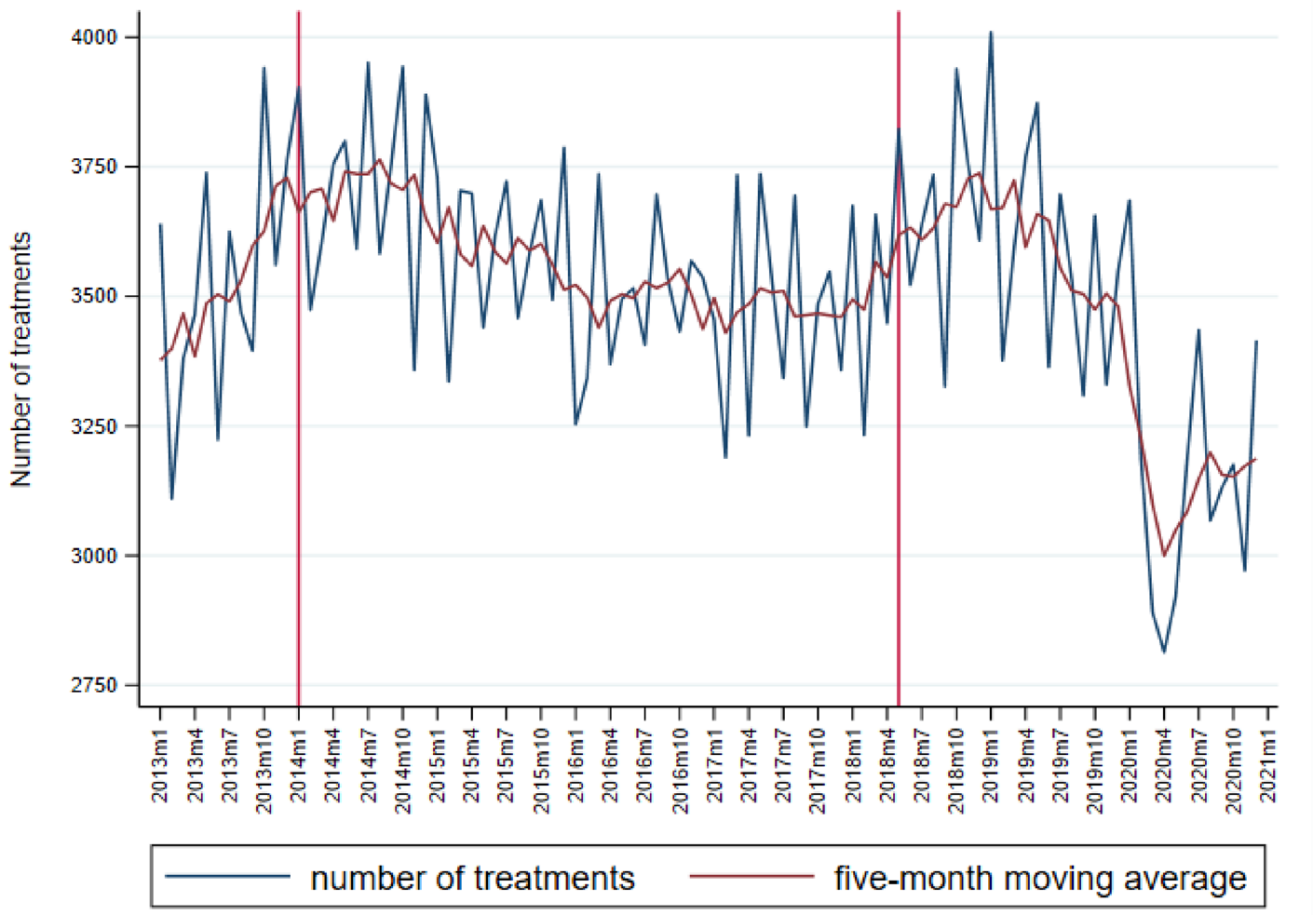
Total number of trastuzumab treatments per month 2013–2019, reference line on January 2014 (introduction subcutaneous Herceptin^®^) and June 2018 (introduction first biosimilar)

Figure 1 depicts the development of the number of total trastuzumab treatments per month. It shows an upward trend in the total number of trastuzumab treatments from January 2013 until January 2014. Then it decreases slightly and is steady at around 3500 treatments per month until June 2018. The introduction of subcutaneous Herceptin^®^ in January 2014 (1st reference line) does not seem to influence the total number of trastuzumab treatments. After the introduction of biosimilars in June 2018 (2nd reference line), total use increases until a peak in January 2019. Hereafter, it decreases slightly again to around 3500 treatments. The large drop in trastuzumab treatments around April 2020 coincides with the Covid-19 pandemic.

**Fig. 2:**
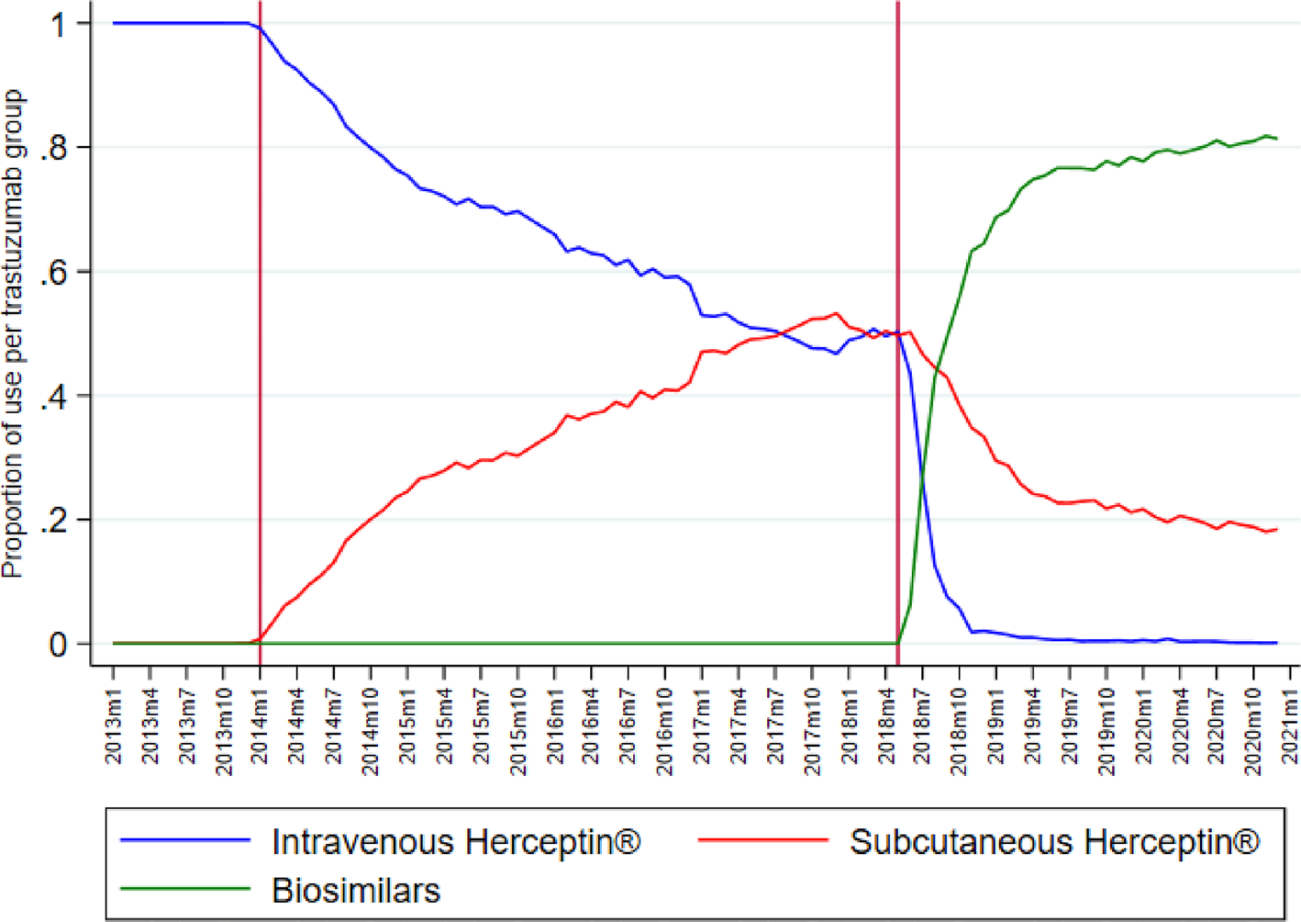
The proportion of trastuzumab treatments per month per administration group 2013–2019, reference line on January 2014 (introduction subcutaneous Herceptin^®^) and June 2018 (introduction first biosimilar)

Figure 2 shows the development of the proportion of the different trastuzumab variants. The introduction of subcutaneous Herceptin^®^ in 2014 leads to a decrease in the use of intravenous Herceptin^®^ and an increase in subcutaneous Herceptin^®^ up to a point in 2017 with a 50%–50% distribution. After June 2018, we see a steep decline in intravenous Herceptin^®^ when the biosimilars are introduced and is barely used anymore a few months later. The proportion of subcutaneous Herceptin^®^ decreases as well but far less steep. In 2020, biosimilars are used for approximately 80% of the treatments and subcutaneous Herceptin^®^ for 20%.

**Fig. 3:**
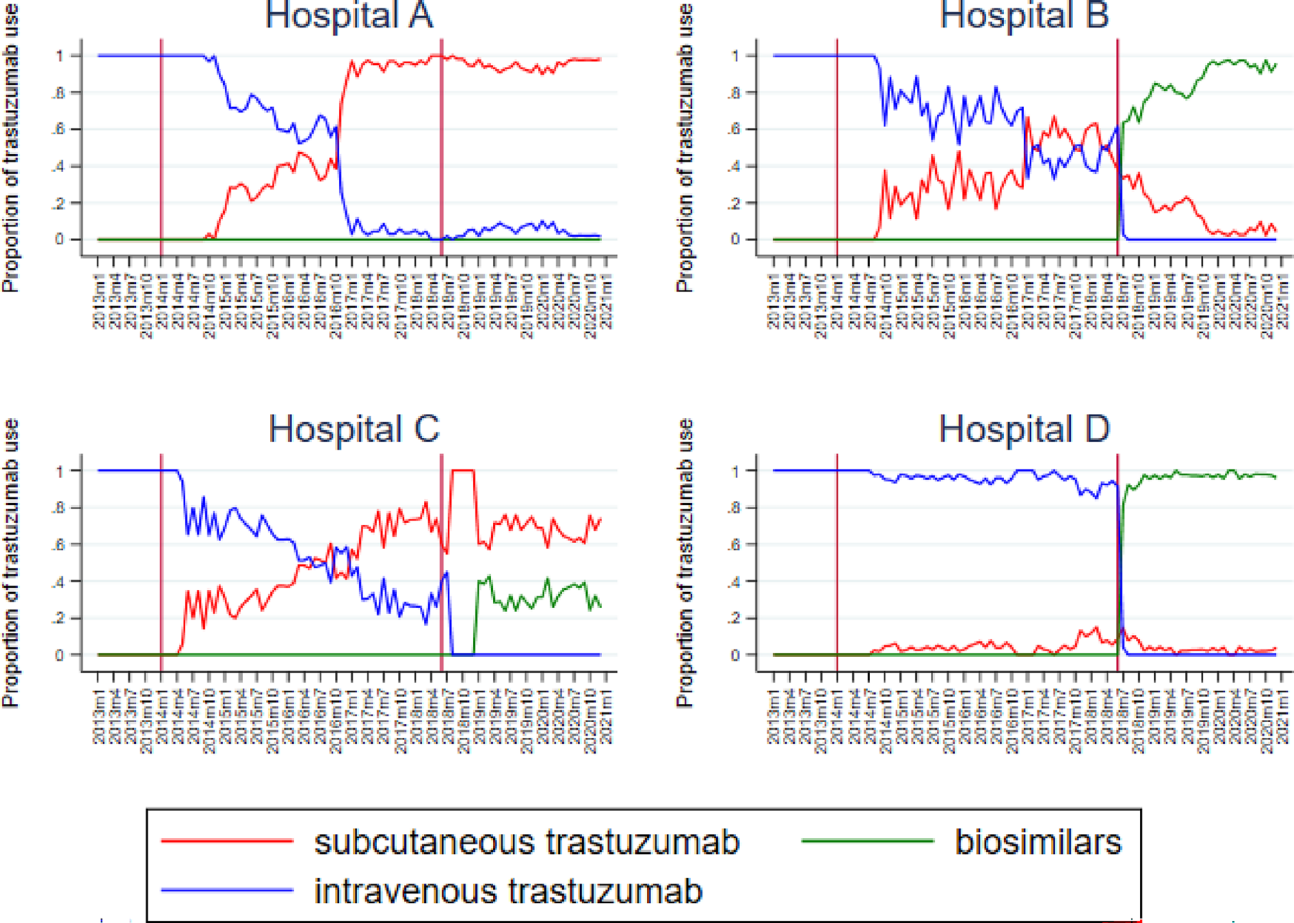
Development of trastuzumab groups for four exemplary hospitals 2013–2019, reference lines on January 2014 (introduction subcutaneous Herceptin^®^) and June 2018 (introduction first biosimilar)

Hospitals have different uptake patterns of both subcutaneous Herceptin^®^ after its introduction in 2014 and biosimilars in June 2018 (Fig. 3). Some hospitals decided to make a full switch from intravenous Herceptin^®^ to subcutaneous Herceptin^®^, whereas some reach an approximate 50%–50% distribution. We also observed differences between hospitals in the uptake of biosimilars. One hospital, with a full switch to subcutaneous Herceptin^®^, decided to keep on using subcutaneous Herceptin^®^ for all treatments, while another hospital switched to using biosimilars for 90% of the treatments. Some hospitals decided not to switch to subcutaneous Herceptin^®^ at all. These hospitals replaced intravenous Herceptin^®^ for biosimilars quickly after its introduction. Hospitals also differed in the speed in which they switched to subcutaneous Herceptin^®^ and biosimilars. Some hospitals used the different administration route of trastuzumab with new patients, while other hospitals switched existing patients from subcutaneous to intravenous trastuzumab or the other way around.

### Analysis of use

#### Proportion subcutaneous Herceptin^®^

Table 5 shows the results of the ITS analysis on the proportion of subcutaneous Herceptin^®^. The introduction of the biosimilars had a direct significant negative effect (*β* = − 0.0454, s.e. = 0.0239) on the proportion of subcutaneous Herceptin®. The biosimilars also led to a significant declining trend (*β* = − 0.0254, s.e. = 0.0030) in the proportion of subcutaneous Herceptin^®^ in the period after the cut-off point. The number of patients treated in a hospital per month positively affects (*β* = 0.0011, s.e. = 0.000) the use of subcutaneous Herceptin^®^. The four largest health insurers appear to have different impact on the use of subcutaneous Herceptin^®^ in a hospital compared to the smaller health insurers. When Insurer A is the dominant insurer in a hospital, the hospitals, on average, have a 22.98 percent point (*β* = − 0.2298, s.e. = 0.0908) lower proportion of subcutaneous Herceptin^®^ than the reference group of smaller insurers. For insurer B this is, on average, a 15.16 percent point (*β* = − 0.1516, s.e. = 0.0575) lower proportion subcutaneous. If the market share of the largest health insurer within a hospital is higher, it results in a higher proportion of subcutaneous Herceptin^®^ usage (*β* = 1.0200, s.e. = 0.1939).

**Table 5:** Interrupted time series analysis results regarding the impact of introduction of biosimilars (2018m6) on the proportion of subcutaneous Herceptin^®^ used on a hospital level

Variables	*β*	SE
Intro biosimilar	− 0.0454*	0.0239
Time (2018m6 = 0)	0.0018*	0.0010
Time^2^	− 0.0001***	0.0000
Intro*Time	− 0.0254***	0.0030
Intro*Time^2^	0.0007***	0.0001
Patients per month	0.0011***	0.0008
Insurer A	− 0.2298**	0.0908
Insurer B	− 0.1516**	0.0575
Insurer C	− 0.0093	0.0732
Insurer D	0.0757	0.0500
Proportion dominant insurer	1.0200***	0.1939
Age	− 0.0015	0.0012
Constant	− 0.0081***	0.0012
*R*^2^	0.2855	

*SE* Standard error

*p < 0.1

**p < 0.05

***p < 0.01

#### Volume effects

After the introduction of the biosimilar, we observe a significant and direct volume effect on the total treatments given in the Netherlands. The number of treatments increased with 197.7657 (s.e. = 125.6392)) treatments at the cut-off point (Table 6). However, we see a negative post-introduction trend relative to the pre-introduction trend (*β* = − 31.3030, s.e. = 15.5631). The increase in treatments is caused by a relative strong increase by hospitals that (predominantly) use the subcutaneous from (see Appendix B, Table 11).

**Table 6:** Interrupted time series analysis results regarding the impact of introduction of biosimilars (2018m6) on the total number of treatments and patients per month for intravenous trastuzumab

Variables	Total number of treatments (*n* = 84)		Patients per month (*n* = 84)	
	*β*	SE	*β*	SE
Time since study	6.1242	5.2754	5.4036***	1.9147
Intro biosimilar	197.7657	125.6392	130.0803**	51.6029
Intro*Time	– 31.3030**	15.5631	– 12.5455*	6.6012
Time^2^	0.2021**	0.0973	0.0373	0.0372
Intro*Time^2^	0.5835	0.6439	0.0732	0.2685
Covid-19	– 511.5575***	173.7247	– 258.5690***	71.0989
Constant	3214.8920***	227.2946	2118.3730***	82.7837
*R*^2^	0.4640		0.8674	

Analyses were performed on a national level

*SE* Standard error

*p < 0.1

**p < 0.05

***p < 0.01

The number of patients in all hospitals increased significantly in the time up to the biosimilar introduction (*β* = 5.4036, s.e. = 1.9146) as well as after the biosimilar introduction with 130.0803 patients (s.e. = 51.6028). Also here, ‘subcutaneous’ hospitals treat more patients than the ‘biosimilar’ hospitals (see Appendix B, Table 12).

In all the analyses, we see that the Covid-19 epidemic negatively affected the total treatments per month and the mean number of patients treated per month. The ‘subcutaneous’ hospitals seem to be less effected by Covid-19.

Lastly, we looked at whether the dosage (based on patient’s weight) for intravenous trastuzumab (intravenous Herceptin^®^ and biosimilars) changed after the introduction of biosimilars (Table 7). While there already was an increasing trend (*β* = 0.6044, s.e. = 0.0864) in milligrams per dosage pre-introduction, the dosage increased with 10 mg (s.e. = 4.1277) post-introduction.

**Table 7:** Interrupted time series analysis results regarding the impact of introduction of biosimilars (2018m6) on the dosage strength for intravenous trastuzumab

Variables	MG dosage intravenous trastuzumab	
	*β*	SE
Time since study	0.6044***	0.0864
Intro biosimilar	10.0305**	4.1277
Intro*Time	0.0239	0.1361
Constant	393.2895***	2.7234
*R*^2^	0.8810	

Analyses were performed on a national level

*SE* Standard error

*p < 0.1

**p < 0.05

***p < 0.01

#### Descriptives of costs

**Fig. 4:**
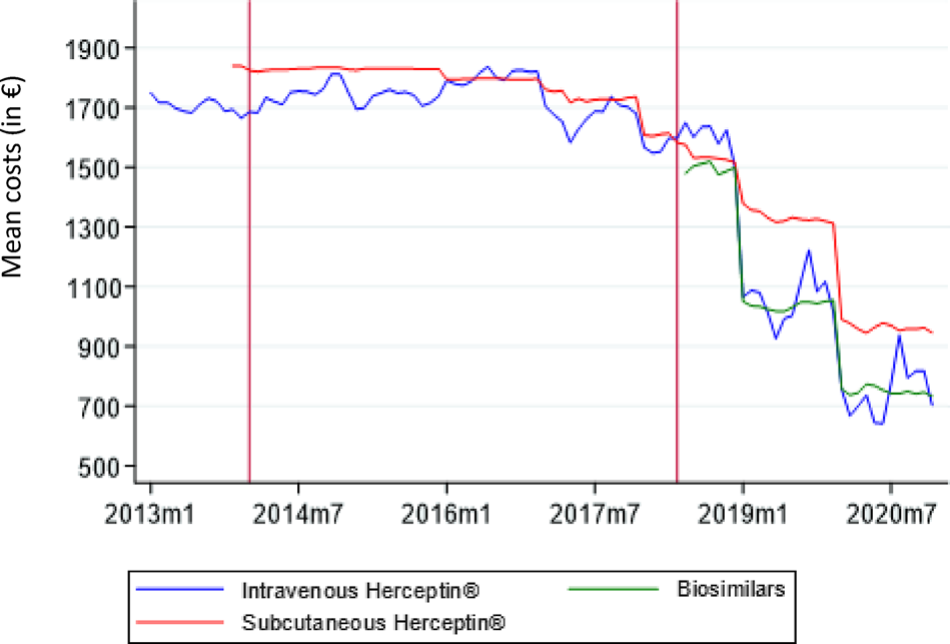
Development of costs per trastuzumab group 2013–2019, in euros

We will make an initial estimation of the public healthcare costs of this evergreening strategy from an insurance (payer) perspective. As the administration costs are reimbursed the same regardless the administration form (intravenous versus subcutaneous) or location of administration (hospital versus at home), we do not include these costs in our analysis. Table 8 and Fig. 4 show the development of the costs per trastuzumab variant. The mean insurer costs for intravenous Herceptin^®^ and subcutaneous Herceptin^®^ are €1718.27 (s.e. = 721.12) and €1620.66 (s.e. = 334.77), respectively. Biosimilars have mean costs of €987.97 (s.e. = 462.25). Subcutaneous Herceptin^®^ enters the market with higher costs than intravenous Herceptin^®^ and it remains higher for all years, except 2016. The biosimilars have mean costs which are lower than subcutaneous Herceptin^®^ for all years and decrease over the years. The costs for subcutaneous Herceptin^®^ substantially decrease with the introduction of the biosimilars. As is clear from Table 8, the average cost of trastuzumab in the biosimilar period is about 48% lower than in the patent period, in 2020 even 57%. After the introduction of the biosimilars also the costs of subcutaneous Herceptin^®^ (still under patent) substantial dropped; however, the drop in costs is about 34%.

**Table 8:** Descriptives of mean add-on reimbursement costs per trastuzumab group in euros per year

	*M*	2013	2014	2015	2016	2017	2018	2019	2020
Herceptin IV^®^	1718.27 (721.12)	1704.69 (731.04)	1740.55 (746.56)	1733.11 (740.63)	1805.21 (745.84)	1674.80 (640.91)	1588.08 (596.20)	1053.67 (314.21)	732.27 (348.43)
Herceptin SC^®^	1620.66 (334.77)		1830.99 (40.95)	1829.39 (40.96)	1795.24 (93.76)	1734.03 (309.05)	1567.11 (249.46)	1335.18 (257.57)	964.65 (293.98)
Biosimilar	987.97 (462.25)						1496.65 (539.62)	1037.97 (355.31)	748.88 (349.184)

Standard deviation in parentheses

With a mean of 18 treatments per patients, this leads to intravenous trastuzumab treatment costs per patient in the period 2013–2017 of €31,170.10 for intravenous Herceptin^®^, €32,353.43 for subcutaneous Herceptin^®^ and €16,081.65 for biosimilars per patient in the period 2019–2020.

Based on these differences in costs between subcutaneous Herceptin^®^ and biosimilars, forgone savings in costs using subcutaneous Herceptin^®^ were estimated in two scenarios. First, if all treatments were substituted with biosimilars from June 2018 onwards, and all treatments were claimed at the average biosimilar costs, €5.4 million could have been saved on drug expenditures in the period June 2018 until December 2020 compared to the situation as is. Current total costs in the period June 2018–December 2020 are €110.8 million. This is a 4.9% increase in costs compared to a situation in which there would have been a 100% switch to biosimilars. Second, if the evergreening strategy would have been fully successful, i.e., the biosimilars would not have entered the market, the societal cost of would have been € 178.5 million compared to the situation as is.^9^

## Discussion and conclusion

### Main findings

In this paper, we explored the dynamic market share and public healthcare costs of trastuzumab’s evergreening (subcutaneous) variant during introduction of trastuzumab’s competitive biosimilar variant in the Netherlands. Our analysis showed that market share of Subcutaneous Herceptin^®^ grew from 0% at introduction in 2014 to 50% in 2017, and due to the introduction of biosimilars in 2018 declined to 20% by 2020. Second, we found that the introduction of subcutaneous administration form did not change the price level of trastuzumab, whereas the introduction of biosimilars was accompanied by lowering price effects associated with biosimilars. Third, we found an increasing volume effect after the biosimilar introduction. Fourth, the switching decision is made on the hospital level and is influenced by patient volume. As the switching decision is made at the hospital level it seems that the switching decision is unrelated to patient preferences. Finally, we found health insurer specific effects in the use of subcutaneous Herceptin^®^.

#### Market share and price effects

The introduction of subcutaneous trastuzumab leads to a gradual uptake, which declined after the introduction of the (intravenous) biosimilars two years later. Probably, the uptake of the subcutaneous form was already influenced by the notion that biosimilars soon would enter the market. Within the hospitals, there are three possible explanations for the gradual decline of the use of the subcutaneous form: 1) hospitals only treat new patients with biosimilars, 2) depleting existing subcutaneous supply or respecting (annual) contracts, and 3) hospitals may anticipate on further price decreases of biosimilars. Shortly after the first biosimilar, pharmaceutical companies launched several other biosimilars. Hospitals could have decided to wait for the second or third biosimilar, because the price can be expected to decrease further due to increased competition. This is also visible in the development of the costs for all three trastuzumab groups which decreased substantially at the beginning of 2019 and 2020.

Striking is the development of the price level of trastuzumab. Only after the introduction of biosimilars the prices decrease. This price decrease concerns not only the intravenous form but also the price of the patent-protected subcutaneous form. Roche’s strategy partly disrupted the competitive biosimilar market. Based on reimbursement costs in the claims dataset, this strategy resulted in an additional societal costs of €28 million.^10^

#### Volume effects

Lower prices due to the introduction of biosimilars can also lead to an increasing volume effect negating the savings. Our results show that the total number of treatments increased significantly on a national level as well as the number of treated patients at the point of biosimilar introduction. A striking observation is that the number of patients increased more for ‘subcutaneous’ hospitals than for ‘biosimilar’ hospitals, an effect that we would expect to happen the other way around given the lower prices for biosimilars. Are the logistic and practical issues of more impact? Are patients more willing to start treatment when it can be administered subcutaneously? Moreover, we found that only ‘subcutaneous’ hospitals have a significant increase in the total number of treatments. However, it must be stated that the analysis for these volume effects was based on only five hospitals; therefore, outliers could have had a magnifying effect on the regression coefficient. Our findings are consistent with the study by Müskens et al. [6, 7], which found that the reduction in expensive medicine prices was accompanied with an increased utilization of these expensive medicines. Although this results in less savings than anticipated, it may lead to better treatment access for more patients. From a medical perspective, it is unclear whether there was undertreatment before or overtreatment after the introduction of the biosimilars.

Another volume effect which may lead to less intended savings is the observed increase in dosage strength at the introduction of biosimilars. The dosage strength for intravenous trastuzumab already increased significantly in the period before biosimilar introduction. A possible explanation for this could be that patients are getting heavier over time, demanding a higher dosage for intravenous trastuzumab. After the biosimilar introduction, dosage strength increased significantly with 10 mg.^11^ Another possible reason for this increase could be spillage caused by the lower price of biosimilars, which would decrease potential savings.

Roche’s strategy led to an estimated 5% increase in medicine costs compared to a situation in which biosimilar uptake was not disrupted and all hospitals made a complete switch to biosimilars. Based on reimbursement costs in the claims dataset, the strategy could have generated in the Netherlands a revenue of an additional €28 million for Roche after biosimilar introduction.^12^

#### Hospital-level switching decisions

Looking at a hospital level, we saw that not all hospitals decided to switch to subcutaneous Herceptin^®^. The majority of hospitals made a dichotomous decision: a complete switch to subcutaneous Herceptin^®^ or staying with intravenous Herceptin^®^. The costs based on insurance claims did not significantly differ between intravenous Herceptin^®^ and subcutaneous Herceptin^®^ with the costs of subcutaneous administration being a bit higher. Literature suggests that subcutaneous administration of trastuzumab is preferred by healthcare providers and patients as the administration takes less time and allows for treatment at patients’ homes [28, 39]. In their decision to switch or not, hospitals may have been aware of the upcoming patent expiry of intravenous Herceptin^®^ and the anticipated biosimilars, and therefore decided to keep on using intravenous Herceptin^®^. This results in only one switch period and makes the switch to biosimilars later on easier as the switch from intravenous Herceptin^®^ to the intravenous biosimilar will be more accepted by patients [36]. Conversely, the evergreening strategy of Roche has the effect that hospitals that switched to subcutaneous Herceptin®, and subsequently to biosimilar have twice the switching costs.

Due to the larger patient volume in general and top clinical hospitals, using the subcutaneous trastuzumab variant with shorter administration time may be driven by logistical considerations. The number of treated patients per month has an increasing effect on the proportion of subcutaneous Herceptin^®^. Hospitals with more patients might experience higher workload on the oncology daycare ward and using subcutaneous trastuzumab may relieve some of this pressure due to shorter administration time and lower costs for nurse time [40]. That is very much understandable if one takes into consideration the (lack of) flexibility of the hospital to accommodate intravenous versus subcutaneous administration. Because of the many new IV-long-term infusions in oncology, there can be a shortage of staff and treatment-space in certain hospitals. Probably, the staff shortage will have a more prominent role in the upcoming years in the Netherlands. Then, SC treatment is a solution for that, whatever the cost. However, this is only the case when subcutaneous Herceptin^®^ is administered in the hospital. A major advantage of subcutaneous Herceptin^®^ is that it allows for home treatment. But a study by Franken et al. [32] shows that home-based treatment almost triples the time invested by healthcare professionals compared to hospital-based treatment, which reduces the cost-effectiveness of at home subcutaneous treatment.

#### Patient perspective

The subcutaneous administration has some advantages over the intravenous administration from a patient perspective [39]. Though it is known that patient’s preferences differ [41], it is an open question how much a society is willing to pay extra for patient preference and convenience. This is especially relevant as more pharmaceutical companies of reference biologic medicines patented a subcutaneous administration form before the patent expiry of the intravenous version. Interestingly, in the current situation, the decision for subcutaneous Herceptin^®^ or biosimilars seems unrelated to patient preferences or societal deliberations, but it is based on individual hospital policies, yet the additional costs are borne by all Dutch citizens.

#### Insurers

The role that insurers, the payers in the Dutch health care system, may have on the biosimilar adoption decisions by hospitals is rather unclear and may vary between one insurer and the other. This is driven by their reimbursement policy, usually negotiated annually. Our results suggest that these insurer policies can matter in the uptake of biosimilars. In addition, the proportion of the dominant health insurer’s market share in a hospital had an increasing effect on the use of subcutaneous Herceptin^®^. This can possible be explained by the fact that insurers with a larger market share in a hospital are more dependent on the hospital to provide an appropriate care offer for its insured persons [33, 42]. Therefore, the insurer may be less able to carry out its preference policy and the hospitals’ policy is dominant.

### Strengths, limitations, and recommendations

This research shed some light on the biosimilar uptake among hospitals and the dynamics of evergreening, a strategy which pharmaceuticals are likely to use in the future [4]. A strength of this research is that it used data covering all hospitals in the Netherlands treating patients with HER2 + breast cancer with trastuzumab over the period 2013–2020. We were able to assess and research both the uptake of subcutaneous Herceptin^®^ and the biosimilars nationwide. This in contrast to an earlier study by Müskens et al. [7] which uses data of a single hospital. In the upcoming years, a number of expensive biologics, such as pertuzumab (Perjeta^®^) and ramucirumab (Cyramza^®^), are nearing patent expiration, thus are potential candidates for an evergreening strategy by pharmaceutical companies. Also subcutaneous forms of several immune checkpoint inhibitors (nivolumab, pembrolizumab, atezolizumab) are expected.

This study has two limitations. First, the financial impact of the evergreening strategy is difficult to determine. Using the price difference between intravenous and subcutaneous trastuzumab as representation of the additional costs paid for subcutaneous Herceptin^®^, will be an underestimation. These costs only reflect what the health care insurer (the Dutch payers) will pay the hospitals but naturally, it does not reflect what the hospital actually pays the pharmaceutical company. Actual purchase prices are mostly confidentially negotiated and therefore not publicly available, also in this case. Hospitals can put a margin on the purchase price for a drug or cross-subsidize it with other hospital products. It could be the case that the difference between the purchase prices of both trastuzumab forms is larger or smaller than the €200–€300 difference found in this study. A larger difference seems more likely since it needs to be financially attractive for hospitals to invest in the switch from subcutaneous Herceptin^®^ to biosimilars; the switching costs can be offset by savings in nursing costs [40]. Moreover, it is unknown how the competitive price for trastuzumab would have developed without the introduction of a new patented administration form. Our cost estimations should therefore be interpreted with caution. Second, using claims data we could not differentiate between the actual costs of administering IV and SC. While these differences can be very relevant for the behavior of the hospitals, in the Dutch system of hospital bargaining these differences have no effect on public spending (which is mainly determined by the bargaining between insurers and hospitals [33]). Decisions such as choosing an administration form are considered to be business decisions made by the hospital.

Further research is needed on the impact of pharmaceutical strategies nearing patent expiration [4] on the uptake of biosimilars and the public healthcare costs. These future studies should focus on other expensive medicines (in other medical specialties) and other strategies employed by pharmaceutical companies. To capture the full societal costs, a comparison between the patient’s opportunity costs due to administering biosimilars and subcutaneous patented drugs and the extra switching costs should be included in future studies, as well as differences in administration costs. Additionally, further research is needed to investigate whether there are volume effects of biosimilar introduction and if so, why these volumes change and whether these volume changes are the effect of undertreatment before the biosimilar introduction or overtreatment thereafter.

Furthermore, as it seems that the choice for intravenous biosimilars or subcutaneous administration form in the current study is based on individual hospital policies rather than on patient needs, it is important to investigate the reasoning behind these policies. As the costs are paid by society, we recommend a more explicit societal debate to consider if the potential benefits of subcutaneous Herceptin^®^ (and other similar medicines) are worth the additional costs. These policies can differ between countries and other medical specialties since there exist different attitudes toward the use of biosimilars across countries and medical specialists [42–44]. In addition, in budget systems, differences in administration costs can be very relevant in choosing which administration form is preferred by the hospital.

## Conclusion

We found a high biosimilar uptake for trastuzumab in the Dutch market, resulting in a more competitive market structure for trastuzumab resulting in significant price drops. The introduction of trastuzumab biosimilars lowered the use of subcutaneous Herceptin^®^. Intravenous Herceptin^®^ is completely substituted with biosimilars after its introduction. A full switch to biosimilar was, however, not made. Ultimately, subcutaneous Herceptin^®^ retained a 20% market share after biosimilar introduction. Additionally, there was an increase in the number of treatments and the number of patients after biosimilar introduction, possibly due to lower prices of biosimilars, indicating that biosimilars can help managing budget constraints.

Given the significant difference in price between subcutaneous Herceptin^®^ and biosimilars the evergreening strategy of pharmaceutical companies near patent expiration leads to lower cost savings for society. Trastuzumab is not the only expensive medicine for which an ‘subcutaneous’ evergreening strategy was used and it is expected this evergreening strategy will be used more frequently in the future since other biological drugs are reaching their patent expiry. As the costs are publicly funded, we recommend a more explicit societal debate to consider if the potential benefits of subcutaneous Herceptin^®^ (and other similar medicines) are worth the additional costs, and at which price it should be reimbursed as the part of the benefit package.

## Figures and Tables

**Fig. 5:**
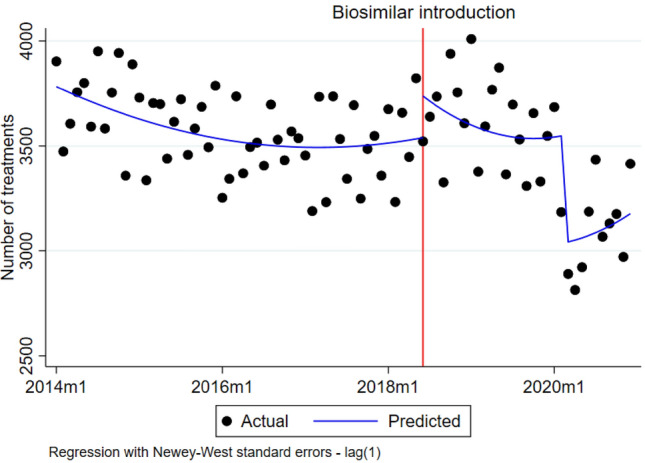
The impact of the introduction of biosimilars on the total number of treatments

**Fig. 6:**
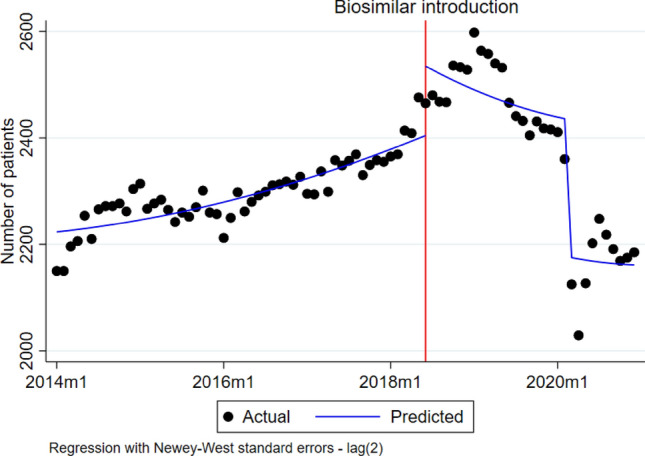
The impact of the introduction of biosimilars on the total number of patients

**Fig. 7:**
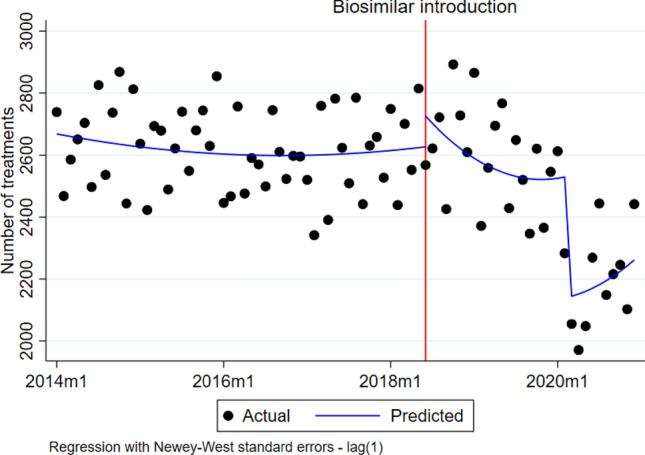
The impact of the introduction of biosimilars on the total number of treatments in biosimilar hospitals

**Fig. 8:**
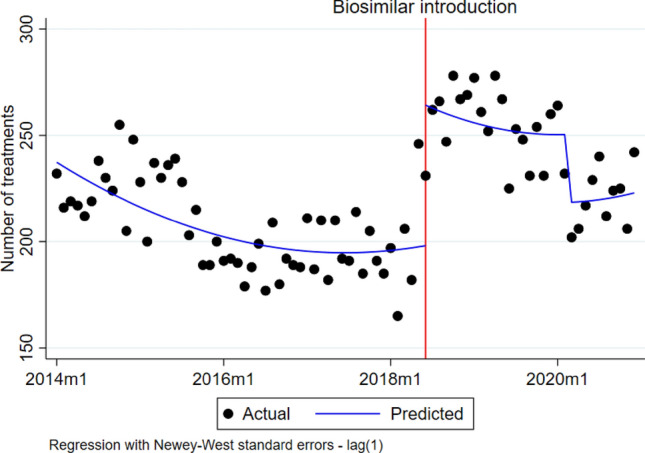
The impact of the introduction of biosimilars on the total number of treatments in subcutaneous hospitals

**Fig. 9:**
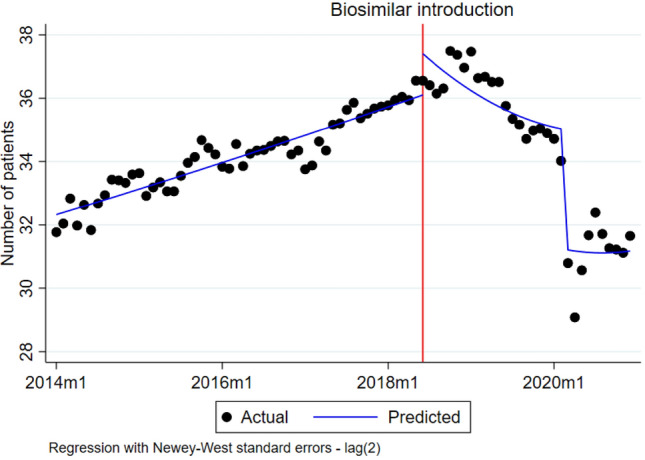
The impact of the introduction of biosimilars on the total number of patients in biosimilar hospitals

**Fig. 10:**
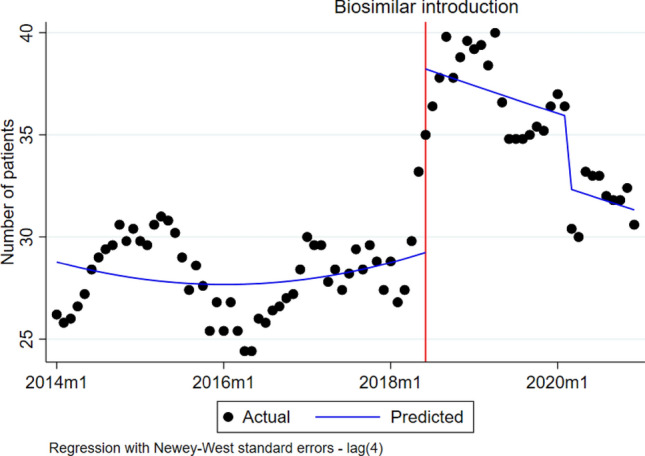
The impact of the introduction of biosimilars on the total number of patients in subcutaneous hospitals

**Fig. 11:**
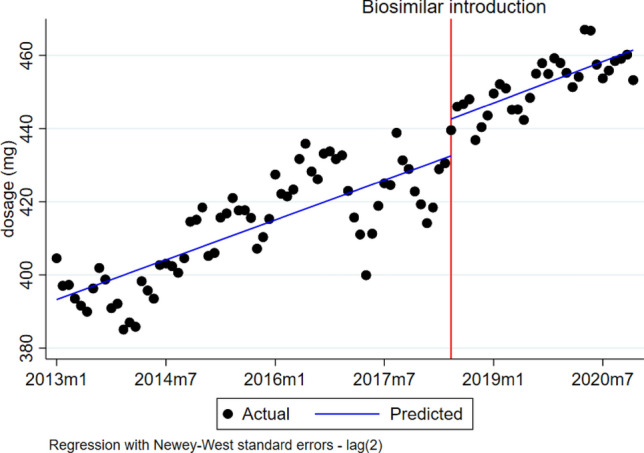
The impact of the introduction of biosimilars on the dosage strength (mg) for intravenous trastuzumab

## Appendix A

### Interrupted time series analysis without simultaneous chemotherapy

Table 9 shows the results of the ITS analysis, excluding patients receiving simultaneous chemotherapy, on the proportion of subcutaneous Herceptin^®^. Results are similar to the analysis including patients receiving simultaneous chemotherapy, indicating that the choice for subcutaneous or intravenous trastuzumab is mainly made on a hospital level and not at the patient level.

**Table 9:** Interrupted time series analysis results regarding the impact of introduction of biosimilars (2018m6) on the proportion of subcutaneous Herceptin^®^ used on a hospital level

Variables	*β*	SE
Intro biosimilar	− 0.0375	0.0258
Time (2018m6 = 0)	0.0010	0.0010
Time^2^	− 0.0002***	0.0000
Intro*Time	− 0.0285***	0.0034
Intro*Time^2^	0.0008***	0.0001
Patients per month	0.0018***	0.0009
Insurer A	− 0.2332**	0.0989
Insurer B	− 0.2208***	0.0680
Insurer C	− 0.0186	0.0924
Insurer D	0.1140*	0.0592
Proportion dominant insurer	1.2212**	0.1991
Age	− 0.0013	0.0012
Constant	0.1083	0.1336
R^2^	0.3092	

*SE* Standard error

**p* < 0.1

***p* < 0.05

****p* < 0.01

## Appendix B

### Interrupted time series analysis volume effects

To test whether the number of treatments are related to the uptake of biosimilars, we assessed whether there was a difference in increase between biosimilar hospitals and those who use subcutaneous Herceptin^®^. Hospitals were classed into nine categories based on their trastuzumab use before (*t* = 0) and after (*t* = 1) the introduction of the biosimilars. Hospitals were classed as subcutaneous hospitals in *t* = 0 when subcutaneous proportion was equal to or exceeded 0.80 in the first two quarters of 2018, and in *t* = 1 when it was equal to or exceeded 0.80 after 2018. Hospitals were classified as biosimilar IV hospitals in *t* = 1 when the biosimilar proportion was equal to or exceeded 0.80. All hospitals not included in these categories were classified as intravenous/subcutaneous trastuzumab hospitals. Table 10 shows the classification of these hospitals.

**Table 10:** Classification of hospitals based on their trastuzumab use

T = 0 T = 1	SC	IV/SC	IV
SC	5	4	9
IV/SC	0	11	24
IV	0	3	16

T = 0 is before biosimilar introduction. T = 1 is after biosimilar introduction

In the analysis, we compare the 49 IV/biosimilar hospitals to the 5 subcutaneous hospitals. There is no significant increase in the number of treatments for biosimilar hospitals (*β* = 98.9412, s.e. = 91.5745) after the introduction, while there is a significant increase for subcutaneous hospitals (*β* = 66.0861, s.e. = 16.2314), see Table 11.

**Table 11:** Interrupted time series analysis results regarding the impact of introduction of biosimilars (2018m6) on the total number of treatments for biosimilar and subcutaneous hospitals

Variables	Biosimilar hospitals (*n* = 49)		Subcutaneous hospitals (*n* = 5)	
	*β*	SE	*β*	SE
Time since study	2.7178	3.6998	0.5766	0.7815
Intro biosimilar	98.9412	91.5745	66.0861***	16.2314
Intro*Time	− 27.2579**	11.6308	− 2.0239	1.4998
Time^2^	0.0661	0.0684	0.0248*	0.0134
Intro*Time^2^	0.6687	0.4765	0.0131	0.0594
Covid-19	− 389.8017***	127.6729	− 31.9547***	11.5920
Constant	2483.0470***	163.1688	167.5523***	32.9279
*R* ^2^	0.4688		0.6478	

*SE* Standard error

**p* < 0.1

***p* < 0.05

****p* < 0.01

There also appears to be a difference in the increase in number of patients between biosimilar and subcutaneous hospitals at the cut-off point (see Table 12). Biosimilar hospitals treated 1.30 ((s.e. = 0.5806) additional patients while for subcutaneous hospitals this is 8.98 ((s.e. = 1.8178) patients. For biosimilar hospitals, the post-introduction trend changed negatively relative to the pre-introduction trend (*β* = − 0.2687, s.e. = 0.0829).

**Table 12:** Interrupted time series analysis results regarding the impact of introduction of biosimilars (2018m6) on the number of patients for biosimilar and subcutaneous hospitals

Variables	Biosimilar hospitals (*n* = 49)		Subcutaneous hospitals (*n* = 5)	
	*β*	SE	*β*	SE
Time since study	0.0764***	0.0154	0.1094	0.1018
Intro biosimilar	1.3004**	0.5806	8.9847***	1.8178
Intro*Time	− 0.2687***	0.0829	− 0.2265	0.1985
Time^2^	0.0001	0.0003	0.0019	0.0019
Intro*Time^2^	0.0036	0.0033	− 0.0018	0.0070
Covid-19	− 3.7817***	0.9981	− 3.5083**	1.5019
Constant	32.0499***	0.7087	23.4441***	4.4608
*R* ^2^	0.8927		0.8216	

*SE* Standard error

**p* < 0.1

***p* < 0.05

****p* < 0.01

Figures from the interrupted time series analysis are presented below. Analysis was performed on a national level, including patients treated with simultaneous chemotherapy. Figure 5 shows the impact of biosimilar introduction on the total number of treatments in the Netherlands over time. Figure 6 shows the impact of biosimilar introduction on the total number of patients in the Netherlands over time.

We performed separate analysis for biosimilar hospitals (*n* = 49) and subcutaneous hospitals (*n* = 5) as well. Figures 7 and 8 show the impact of biosimilar introduction on the total number of treatments in biosimilar and subcutaneous hospitals, respectively.

Figures 9 and 10 Show the impact of biosimilar introduction on the mean number of treated patients in biosimilar and subcutaneous hospitals, respectively.

Finally, Fig. 11 shows the impact of biosimilar introduction on the dosage strength for intravenous trastuzumab. This includes intravenous Herceptin^®^ and biosimilars. Subcutaneous Herceptin^®^ is excluded since this is given as a fixed dosage of 600 mg and, therefore, will not be impacted by time or biosimilar introduction.

## Footnotes

1 In the Netherlands, there are ten health insurers. In 2020, the four major health insurers had a market share of 84,7% [32].

2 Trastuzumab can also be used for treatments of metastatic gastric cancer. These patients have been excluded.

3 Our research design deviates from a regression discontinuity design (RDD). In this research, the discontinuity is a moment in time (market entry) and cannot be interpreted as randomly assigned. Our cross-sectional sites (hospital data) allows for cross-sectional variation in the treatment date.

4 To minimize bias, different functional forms were tested: linear, linear interaction, quadratic, quadratic interaction, cubic and cubic interaction. Based on F-tests and AIC, the most appropriate form was chosen: quadratic interaction. Robustness checks were performed by excluding one month (almost 1%), 5 months (almost 6%) and 10 months (almost 12%) of outer data points [37].

5 The panel data analysis estimates coefficients with fixed effects and with Driscoll and Kraay standard errors to control for autocorrelation and possible cross-sectional dependence and heteroscedasticity [38]. The number of lags is determined by the formula $$m\left( T \right) = {\text{floor}}\left[\kern-0.15em\left[ {\left( {4T/100} \right)^{{{\raise0.7ex\hbox{$2$} \!\mathord{\left/ {\vphantom {2 9}}\right.\kern-0pt} \!\lower0.7ex\hbox{$9$}}}} } \right]\kern-0.15em\right]$$ [39].

6 In our analyses, we include all patients, those with and without simultaneous chemotherapy. We also performed an analysis only including patients receiving monotherapy. The results are similar and can be found in Appendix A.

7 The single-center interrupted time series analysis estimates the coefficients by ordinary least squares regression with Newey-West standard errors to control for serial correlation and possible heteroscedasticity. The number of lags are determined by the Cumby-Huizinga test for autocorrelation.

8 In the Netherlands in March 2020, healthcare was scaled back due to the Covid-19 pandemic resulting in less hospital admissions and treatments.

9 For this calculation, we use the average price of Herceptin^®^ for the period 2014m1–2018m5 multiplied by the volume in the period 2018m6–2020m12.

10 Revenue was calculated as the sum of: mean costs per month x mean proportion of subcutaneous trastuzumab x total number of treatments per month in the period June 2018–December 2020. The costs do not adequately reflect what Roche earns from the sales of subcutaneous Herceptin^®^ as we do not have information on the Roche sales prices.

11 As the prescribed dosage is 6 mg/kg, the patients are in the period June 2018–2020 on average 1.7 kilo heavier than in the period 2013–May 2018.

12 Revenue was calculated as the sum of: mean costs per month x mean proportion of subcutaneous trastuzumab x total number of treatments per month in the period June 2018–December 2020. The costs do not adequately reflect what Roche earns for the sale of subcutaneous Herceptin^®^ as we do not have information on the Roche sales prices.
